# The COVID-19 vaccine procurement and supply chain in the Democratic Republic of Congo

**DOI:** 10.3389/frhs.2025.1681053

**Published:** 2026-02-23

**Authors:** Genèse Lobukulu Lolimo, Yannick Musawu Kabadi, Senait Alemayehu Beshah, Rodrigue Khonde, Aurore Beia, Héritier Makongote, Générose Sumaili, Samuel Kabuya, Joél Bongutu, Daniel Malik Achala, Grace Njeri Muriithi, Elizabeth Naa Adukwei Adote, Elias Asfaw Zegeye, Chinyere Ojiugo Mbachu, John Ele-Ojo Ataguba, Fadima Inna Kamina Yaya Bocoum, Serge Manitu Mayaka, et Éric Mafuta Musalu

**Affiliations:** 1 Department of Health Management and Policy, Kinshasa School of Public Health, University of Kinshasa, Kinshasa, Democratic Republic of Congo; 2 National Emergency and Humanitarian Action Program, Ministry of Public Health, Hygiene and Social Welfare, Kinshasa, Democratic Republic of Congo; 3 African Health Economics and Policy Association (AfhEA), Accra, Ghana; 4 Ethiopian Public Health Institute, Health System Research Directorate, Addis Ababa, Ethiopia; 5 Environmental Health Department, Kinshasa School of Public Health, University of Kinshasa, Kinshasa, Democratic Republic of Congo; 6 Department of Nutrition, Kinshasa School of Public Health, University of Kinshasa, Kinshasa, Democratic Republic of Congo; 7 Expanded Program on Immunization, Ministry of Public Health, Hygiene and Social Welfare, Kinshasa, Democratic Republic of Congo; 8 Community Healthcare Department, Kinshasa School of Public Health, University of Kinshasa, Kinshasa, Democratic Republic of Congo; 9 National Border Sanitation Program, Ministry of Public Health, Hygiene and Social Welfare, Kinshasa, Democratic Republic of Congo; 10 Africa Centers for Disease Control and Prevention, Health Economics and Financing Division, Addis Ababa, Ethiopia; 11 Economics Department, University of KwaZulu-Natal, Durban, South Africa; 12 Department of Community Medicine, University of Nigeria, Enugu Campus, Nigeria; 13 Department of Community Health Sciences, Faculty of Sciences, Rax Rady College of Medicine, University of Manitoba, Winnipeg, MB, Canada; 14 Partnership for Economic Policy (PEP), Nairobi, Kenya; 15 School of Health Systems and Public Health, University of Pretoria, Pretoria, South Africa; 16 Health Sciences Institute, Ouagadougou, Burkina Faso

**Keywords:** COVID-19, DRC, procurement, supply, vaccine

## Abstract

The COVID-19 vaccine has been classified as an ‘essential medicine’, yet shortages and unequal distribution during the pandemic have reignited concerns about vaccine self-sufficiency in Africa. This study examined the mechanisms for acquiring, distributing, and administering existing COVID-19 vaccines in the Democratic Republic of Congo (DRC). A qualitative case study was conducted using semi-structured interviews with 23 key informants selected using reasoned choice, based on their professional roles in vaccine policy, logistics, and implementation. Participants were recruited from public institutions, with most being medical doctors and having experience in vaccination. Data were transcribed and analyzed were transcribed and analysis thematically using Atlas-ti 7.0. The study found that vaccine acquisition in the DRC relied heavily on international donations and multilateral initiatives, with limited national financial contribution. Distribution followed a five-tier supply chain managed by the Expanded Program on Immunization, moving vaccines from Kinshasa to provincial and field offices, then to selected health facilities. The Cold chain limitations, transport issues, and inconsistent vaccine availability challenged the administration. To improve vaccine access and coverage, stakeholders emphasized the need to strengthen logistical infrastructure and promote regional vaccine production. Honoring government commitments to co-finance procurement was also identified as a critical step toward sustainable vaccine supply.

## 1 Introduction

Vaccines, as major medical advances, play an essential role in reducing the spread and effects of infectious diseases (1–3). In response to the COVID-19 pandemic, vaccine development progressed at unprecedented speed: over 270 candidates were developed within the first year, using both existing technologies and novel approaches. The Pfizer-BioNTech, Moderna, and Janssen vaccines were among the first approved by the U.S Food and Drug Administration (FDA) (2–8).

Equitable access to a vaccine depends on large-scale production, global supply chains, product acceptability, and accessibility. The Covid-19 vaccine has been classified by the World Health Organization as an “essential medicine”, reinforcing national and international obligations to ensure access for all (9, 10). However, advance purchases by high-income countries and limited global supply created significant barriers for low-resource countries, including those in Africa (11).

Unlike Western and Asian countries with domestic capacity, African nations, including the Democratic Republic of Congo, have relied heavily on external donations and multilateral initiatives. This dependency exposed vulnerabilities in procurement and distribution systems, leading to frequent stockouts and logistical challenges. While discussions around local vaccine production in Africa are ongoing, this study does not focus on production barriers. Instead, it addresses a more immediate and practical concern: how vaccines were acquired, distributed, and administered in the DRC during the COVID-19 pandemic (1, 3, 12, 13).

The social problem at hand is the limited and inconsistent access to COVID-19 vaccines in the DRC, which hindered immunization efforts. The research gap lies in the lack of detailed, country-specific analyses of the vaccine supply chain, particularly in fragile health systems. Previous studies have examined vaccine hesitancy and global distribution inequities, but few have explored the operational mechanisms within national programs in sub-Saharan Africa (1, 2, 9, 12, 14, 15).

This study aimed to describe the mechanisms for acquiring, distributing, and administering existing COVID-19 vaccines in the Democratic Republic of Congo, and identify logistical and policy-level approaches that could improve equitable access and coverage.

## 2 Methods

### 2.1 Type of study, scope, and period of study

This study employed a qualitative case study design focused on key informants involved in COVID-19 vaccine procurement and distribution in the Democratic Republic of Congo (DRC). Data were collected between September to October 2024 across four provinces: Kinshasa, Ituri, Maniema, and Tshuapa.

### 2.2 Data collection techniques and sources

We conducted semi-structured key-informant interviews in French, using a guide developed for a multi-country project implemented across nine African nations. The guide covered themes such as procurement and distribution mechanisms, local-regional vaccine manufacturing, equity and vulnerable populations, public-private partnerships in immunization, and gender consideration. Interviews were conducted face-to-face with stakeholders directly involved in COVID-19 vaccine management.

### 2.3 Study population, sample size, and participant selection

The study targeted individuals directly involved in vaccine acquisition, distribution, and administration across the three levels of the DRC health system:

- Central level: Ministry of Health, General Secretariat, national programs, and inspectorates
- Intermediate level: Provincial health divisions and EPI coordination/antenna
- Peripheral level: Health zone offices and healthcare facilities

A total of 23 key informants were selected using a reasoned choice (purposive sampling), based on their professional roles and direct involvement in COVID-19 vaccination. The distribution was as follows:

- Central level: 4
- Intermediate level: 10
- Peripheral level: 9

This stratified selection ensured representation across governance levels and geographic diversity. The sample size was determined based on thematic saturation and logistical feasibility.

### 2.4 Quality assurance of collected data

The principal investigator trained a physician and a public health specialist in the study protocol, who then trained two additional interviewers. Interviews were audited progressively to ensure consistency and data quality. Transcripts were anonymized to protect participant confidentiality.

### 2.5 Data analysis

The interviews were recorded and transcribed verbatim. The audios were listened to, and the transcripts were read several times to become familiar with their content. The transcribed interviews were coded and analyzed to identify recurring themes, differences, and unique viewpoints expressed by the participants.

Data analysis was carried out using the framework analysis method, which comprises five stages: (i) familiarization; (ii) identification of a thematic framework; (iii) indexing; (iv) graphic representation; (v) mapping and interpretation (16). The thematic framework was developed using deductive and inductive codes. Thematic analysis was the main analytical approach used in this study. The aim was to systematically and objectively identify, group, and examine the themes arising from the theoretical framework and the objectives of the study (16–18). In addition, new emerging themes were taken into account to reveal the participants’ experiences, while maintaining a critical, neutral, and reflexive approach. Two researchers carefully read the full transcripts of the interviews they were analyzing. Atlas-ti 7.0 software was used for coding and thematic analysis.

### 2.6 Ethics

Transcripts were anonymized during data analysis by removing all participants’ personal information. The research protocol was submitted to and approved by the ethics committee of the Kinshasa School of Public Health under approval number ESP/CE/033/2024.

## 3 Results

### 3.1 Characteristics of respondents

The study included 23 key informants, primarily physicians (13), with most working at the intermediate level of the health system (10). The majority were male (19), aged 50 or older (13), had less than 10 years’ experience in vaccination (12), and were affiliated with public institutions. A detailed breakdown is provided in Table 1.

**Table 1:** Socio-demographic characteristics of study participants.

Characteristics	*n* = 23	%
Gender		
Female	4	17,4
Male	19	82,6
Age		
≤30	1	4,4
31–49	9	39,1
≥50	13	56,5
Respondent's institution		
EPI^1^	3	13,0
Public-sector institutions/structures	13	56,5
Private-sector institutions/structures	3	13,0
Implementing partners/civil society and FTPs^2^	4	17,4
Intervention level		
Central	4	17,4
Intermediate	10	43,4
Operational	9	39,1
Respondents’ professions		
Nurse	3	13,0
Doctor	13	56,5
Pharmacist	1	4,4
Logistician	2	8,7
Other	4	17,4
Years of vaccination experience		
<10 years	12	52,2
≥10 years	11	47,8

^1^ EPI, expanded program on immunization

^2^ FTP, financial and technical partners.

### 3.2 COVID-19 vaccine supply chain and administration in the DRC

#### 3.2.1 Acquisition and procurement

Covid-19 vaccines entered the country through Kinshasa's national warehouse in Kinkole, one of the three national hubs (Kinshasa, Lubumbashi, and Kisangani). During the pandemic, vaccines were acquired from multilateral partners (e.g., COVAX, Gavi, WHO, UNICEF) and bilateral donors (e.g., France, Belgium, the UK, the USA). In 2024, procurement shifted to include government co-financing alongside continued support from GAVI and UNICEF.

From this warehouse, the vaccines are distributed to the 26 provinces, managed by the various coordination (8) and Antennas (51) of the Expanded Program on Immunization (EPI), according to a pre-established distribution plan.

« The COVID-19 vaccines go through UNICEF. Donations have been received from France, Belgium, the UK, Africa CDC, etc.» KI 19

#### 3.2.2 Distribution mechanism

**Figure 1:**
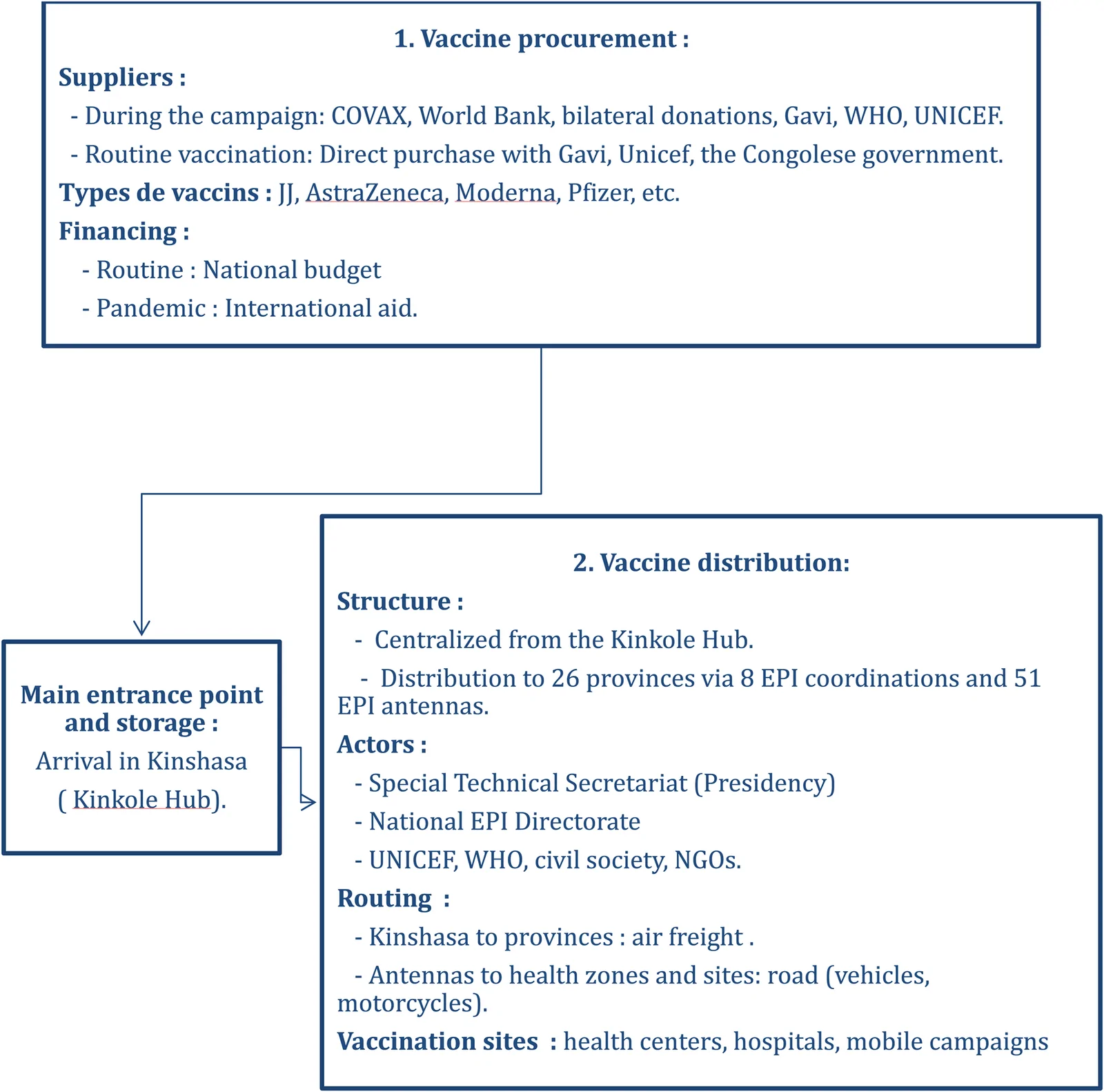
COVID-19 vaccine procurement and distribution flow in the DRC.

The vaccine distribution system followed a five-tier structure: national warehouse –provincial coordination—EPI antennas—health zones –health facilities. The Technical Secretariat under the Presidency coordinated the national response, working closely with the EPI Directorate. Distribution to provinces was primarily by air, while transport to health zones and facilities relied on road or motorcycle, often in partnership with NGOs. (Figure 1).

« The Covid-19 vaccine followed the standard distribution process… provinces are supplied from the central level…» KI 23

« Kinshasa supplies the branches and coordination offices, which supply the health zones, which in turn distribute to the health centers where vaccinations are carried out. » KI 19

#### 3.2.3 Administration process

In the DRC, only selected hospitals and health facilities served as COVID-19 vaccination sites. At the operational level of the Health zone, vaccines were received by zone authorities and managed by EPI supervisors, who distributed them to health facilities and hospitals based on requisitions, maintaining safety margins and cold-chain requirements.

« We compile health facilities requisitions, with a safety margin. When the Antenna supplies us, we also supply the health facilities where there is cold chain equipment. » KI 22

### 3.3 Challenges in the vaccine supply chain

#### 3.3.1 Availability and stockouts

Stockouts were a recurring issue, driven by public hesitancy, short vaccine shelf life, and poor alignment between supply and actual demand. Two-dose vaccines (Pfizer, Moderna) were more effective than single-dose vaccines (Johnson & Johnson). Delays in delivery and weak forecasting further exacerbated shortages.

##### 3.3.1.1 Vaccine out-of-stock

The availability of COVID-19 vaccines in the DRC has been affected by a series of chronological problems. Initially, public reluctance and impending expiry led to the redistribution of a batch of vaccine to other African countries. Then, between 2021 and 2024, batches were destroyed due to their expiry date. Vaccination routinization in 2024–2025 faced stock-outs, mainly due to the high cost of vaccines and dependence on government funding.

Since the last batches of the COVID-19 vaccine in the country expired in December 2023, many Health Zones continue to face stock-outs. Vaccine stock-outs at the health zone level have varied according to beneficiary preferences. Although all four main types of vaccine (AstraZeneca, Pfizer, Moderna, and Johnson & Johnson) were available, Pfizer and Moderna vaccines, requiring two doses, were the most affected by stock-outs, unlike Johnson & Johnson (single dose).

« Well, we ran out of vaccine at times, so we could ask for so much vaccine and only receive half..» KI 13

« You'll see that you've vaccinated a person with an antigen, you'll give them the exact deadline for coming to take the second dose of antigen, and now there’s no second dose of this antigen, so what should we do..» KI 8

##### 3.3.1.2 Low vaccine consumption

Among the reasons for low uptake cited by participants were the rapid expiry of certain batches of vaccine, and the failure to take account of requisitions from healthcare establishments when supplying vaccine to vaccination facilities. The multiplicity of vaccines, with a preference for certain vaccines and low acceptance of the vaccine by the community for fear of its side effects.

« People preferred certain vaccines to others, such as Johnson and Johnson, because they were single-dose. And foreigners preferred Pfizer for its efficacy. This preference led to the expiry of other types of vaccine. » KI 16

##### 3.3.1.3 Vaccine supply delay

Some respondents mentioned delays in vaccine supply as a reason for low vaccine availability, which could explain the low vaccine coverage in Covid-19 vaccination. The country was not regularly supplied with vaccine according to demand, and this had a direct impact on the supply of vaccine at vaccination posts and sites. Another reason for the delay was a lack of forecasting or planning for this vaccine.

« The second difficulty is that sometimes we're not served at the right time. You place the order, the need is there, but the delivery doesn't respect your request. » KI 22

#### 3.3.2 Cold chain

Cold chain infrastructure was insufficient in many regions, particularly remote areas. Shortages of isothermal boxes, accumulators, refrigerators, and refrigerated vehicles compromised vaccine quality. Electricity instability also hindered storage.

##### 3.3.2.1 Low logistics capacity

The logistical capacity of cold chain equipment to ensure the preservation and quality of the vaccine right up to the site of use is low in some parts of the country, especially in hard-to-reach areas. These include isothermal boxes, accumulators, refrigerators, and refrigerated vehicles for transporting vaccines. The quality of the country's existing cold chain also remains a concern.

« Isothermal containers were lacking, and there was also the problem of accumulators and cold storage. Finally, there was even a shortage of refrigerated vehicles for transporting vaccines to the central offices of health zones and health facilities. » KI 14

##### 3.3.2.2 Low electricity coverage

Lack of electricity or untimely power cuts in some parts of the country make it difficult to store the vaccine at the temperatures required to preserve its quality.

« In our country, the big problem is electricity for vaccine storage, and that’s not just for COVID-19. » KI 6

#### 3.3.3 Transport and accessibility

Transport challenges included limited vehicles, high costs, lack of fuel, and poor road infrastructure. Riverine populations required specialized transport, such as motorized canoes.

« I think the big problem is transportation..getting vaccines out of Kinshasa is a real problem …transportation is expensive, and since it’s by plane… » KI 6

#### 3.3.4 Equity in vaccine distribution

Vaccination was uniformly implemented across all health zones. Some zones were prioritized, while others, particularly rural or underserved areas, were excluded, resulting in inequitable access (Table 2).

**Table 2:** Challenges in the COVID-19 vaccine supply chain in Democratic Republic of Congo.

Barrier	Description	Illustrative quote
Availability	Stockouts, expiry, poor forecasting, and low uptake	“We often ran out of vaccine…” — KI 13
Cold chain	Inadequate equipment and unreliable electricity	“We lacked isothermal containers…” — KI 14
Transport	Limited vehicles, poor roads, high costs, and riverine access issues	“Transport is expensive… especially by plane.” — KI 6
Equity	Uneven distribution across health areas	“Some areas were selected; others were not.” — KI 23
Solution	Government commitment, local production, logistics upgrades, PPPs	“Africa should have its own vaccines.” — KI 5

« Some health areas that were selected, but not all… populations in need didn’t receive vaccines…» KI 23

### 3.4 Stakeholders’ recommendations and proposed solutions

#### 3.4.1 Improving availability

Informants emphasized the need for the government to uphold its financial commitments to vaccine procurement. While no local manufacturing initiatives were identified, most respondents supported the idea of domestic production to improve availability and public trust. Continental efforts led by the African Union were also mentioned.

« Africa for Africa..they advocate harmonizing regulations and promoting local production. » KI 5

#### 3.4.2 Strengthening logistics and cold chain

Recommendations included acquiring refrigerated aircraft, trucks, and motorized canoes, using drones and indigo for remote delivery, and opening secondary entry points. The community approach has also been suggested as a way of transporting vaccines. A qualification plan to assess equipment reliability was also recommended.

This involves using the community itself to transport vaccines to hard-to-reach locations. The opening of new entry points for vaccines and the establishment of an intersectoral approach to problem-solving were also proposed.

« We should even have refrigerated aircraft and refrigerated trucks to transport vaccines in good conditions. » KI 1

#### 3.4.3 Enhancing public-private partnerships

Private sector actors were seen as valuable partners in raising awareness, providing logistical support, and contributing financially. However, challenges included limited access to cold chain equipment, and reliability was also suggested.

« We need to make cold chain equipment available to private facilities. » KI 15

Immunization shouldn't just be for the public sector; we also need the private sector to get involved. Some have suggested that the government should exempt certain taxes in terms of public-private partnerships to facilitate their integration into immunization activities.

« The Ministry of Health should offer advantages so private centers can participate. » KI 11

## 4 Discussion

This study aimed to explore the mechanisms for acquiring, distributing, and administering COVID-19 vaccines in the Democratic Republic of Congo, identifying key challenges and stakeholder-driven solutions. Through qualitative insight from 23 informants, the study contributes to a growing body of evidence on vaccine supply chain dynamics in low-resource settings.

### 4.1 Centralized supply chain: strengths and limitations

The DRC's centralized vaccine supply chain, managed by the Expanded Immunization Program (EPI), enabled national-level coordination but revealed limited responsiveness to local needs. This model mirrors approaches in the other African countries, where centralized systems were leveraged to scale up COVID-19 vaccination (19). However, notable differences exist in terms of logistical capacity, financial resources, and preparedness to manage a mass vaccination campaign targeting the at-risk population, particularly adults, a population traditionally less covered by the EPI (20, 21).

Our findings align with recent studies showing that centralized systems, while efficient for routine childhood immunization, struggle to adapt to emergency mass campaigns targeting broader populations [Adhikari et al. (1); OMS 2021; Williams et al. (20); van Kessel et al. (14)]. The DRC's reliance on air and road transport, coupled with cold chain fragility, underscores the need for more flexible, decentralized logistics.

Legge et al. argue that equitable access to vaccines in a country depends on the vaccine supply system and service delivery, and the government's prioritization of resource allocation for immunization (15).

### 4.2 Key challenges in acquisition, distribution, and administration

The study identified five interrelated barriers:

1. Vaccine availability: stockouts, expiry, and poor forecasting disrupted supply.
2. Cold chain limitations: Equipment shortages and unreliable electricity compromised storage
3. Transport constraints: High cost, poor roads, and limited vehicles hindered delivery.
4. Community reluctance: Misinformation and mistrust reduced uptake
5. Equity gaps: Selective distribution excluded some health zones

These findings echo broader African and particularly low- and middle-income countries, where vaccine access was shaped by infrastructure deficits, donor dependency, and weak planning. The DRC's vast geography and security challenges further amplified these issues (1, 22–24). However, the unpreparedness of immunization systems for an epidemic of this scale was particularly pronounced in countries with fragile health systems and limited resources (24).

The literature on preparing healthcare systems for epidemics emphasizes the importance of investing in logistical infrastructure, building the capacity of healthcare personnel, and putting in place contingency plans to deal with emergencies. However, many countries were unprepared for the COVID-19 pandemic, leading to delays in the roll-out of vaccination and insufficient vaccine coverage (22–25). These challenges were amplified by the very nature of the Covid-19 pandemic, which required the rapid deployment of large-scale vaccination against a backdrop of global vaccine and resource shortages (15, 26, 27). Some authors have also mentioned that in developing countries, the lack of storage equipment, vaccine transport, the cold chain, and the short shelf life of the vaccine are obstacles to vaccine supply (1, 25).

### 4.3 Public-private partnership: opportunities and constraints

Informants highlighted the potential of public-private partnerships (PPPs) to support logistics, outreach, and vaccine delivery. Recent collaborations between Africa CDC and UNICEF have shown that PPPs can strengthen procurement and supply chain systems across the continent (28).

However, our study also revealed integration barriers: private facilities lacked cold chain support and faced regulatory hurdles. To be effective, PPPs must be backed by policy incentives, tax exemptions, and inclusion in national immunization activities.

Dellepia et al, as well as Stevens et al, have highlighted the fact that a good public-private partnership can improve healthcare products and services at both national and local levels, with the involvement of healthcare workers and the community (7, 29–31).

### 4.4 Local vaccine production: promise and feasibility

While no local manufacturing initiatives were identified in the DRC, informants supported regional production to reduce dependency and build public trust. The African Union's goal to produce 60% of Africa's vaccines locally by 2040 reflects this ambition (32). Yet feasibility remains a concern. Regulatory harmonization, quality assurance, and financing are major hurdles. As GAVI and WHO emphasize, local production must be paired with robust infrastructure and long-term investment to be sustainable (32). Different authors support the promotion of approved local production with the support of the international community through technology transfer and South-South technical cooperation as an approach to improve vaccine availability in developing countries facing low vaccine availability (1, 15).

### 4.5 Innovative logistics: drones, indigos, and community models

Proposed solutions like drones and indigos (long-duration cold storage carriers) offer promise for hard-to-reach areas. However, cost, scalability, and maintenance pose challenges.

Community-based model, where local actors deliver vaccines, emerged as a low-cost alternative. While less technologically advanced, they may offer greater sustainability and cultural acceptability in remote regions.

Other authors have stressed the need to acquire minimal logistical resources to ensure vaccine transport and storage (1). They proposed that countries wishing to improve the vaccine distribution chain should adopt best practices in vaccine storage and use, avoiding wastage through temperature control from acquisition to distribution and use in immunization activities (2, 12, 33, 34).

### 4.6 Implications for future preparedness

To strengthen pandemic readiness, countries like the DRC must:

- Invest in cold chain and transport infrastructure
- Build health worker capacity for vaccine management
- Develop contingency plans for emergency deployment
- Foster inclusive PPPs and explore regional production
- Improve community engagement to counter misinformation

Thes priorities align with Africa CDC's 2025–2035 framework for resilient health systems (32, 35).

### 4.7 Study limitations

This study has some limitations. First, the sample size (*n* = 23) limits generalizability. Second, no triangulation with quantitative data was conducted. Future research could expand to include community perspectives and comparative analyses across provinces.

## 5 Conclusion

This study concluded that the COVID-19 vaccine supply chain in Democratic Republic of Congo operates through a five levels structure managed by the Expanded Program on Immunization. Extending from Kinshasa to provincial coordination offices/antennas, health zones, and vaccination sites. While this centralized system facilitated national coverage, it also exposed significant structural and logistical challenges.

Key barriers identified limited vaccine availability, cold chain deficiencies, transport constraint, and inequitable distribution across health zones. These challenges were compounded by community hesitancy and insufficient forecasting, which hindered effective vaccine administration.

To address these gaps, stakeholders emphasized the importance of strengthening the logistical infrastructure, promoting inclusive public-private partnership, and exploring regional vaccine production. These approaches, while promising, require sustained government commitment, regular support, and investment in health system resilience.

This study adds context-specific evidence to the growing literature on vaccine supply chain management in low-resource settings. Its findings underscore the need for adaptive strategies that combine technological innovation, community engagement, and multisectoral collaboration.

However, the study has limitations. First, the sample size (*n* = 23) limits generalizability. Second, no triangulation with quantitative data was conducted. Future research should explore comparative models across provinces and include community perspectives to enrich understanding.

In preparing for future pandemics, it is essential to learn from these experiences. Strengthening vaccine delivery systems, improving equity in access, and building trust through transparent governance will be critical to ensuring timely and effective immunization responses.

## Data Availability

The raw data supporting the conclusions of this article will be made available by the authors, without undue reservation.
